# Ablação por Cateter no SUS: Entre a Evidência Consolidada e o Acesso Limitado

**DOI:** 10.36660/abc.20260287

**Published:** 2026-05-21

**Authors:** Andre d’Avila

**Affiliations:** 1 Hospital SOS Cardio Florianópolis SC Brasil Hospital SOS Cardio, Florianópolis, SC – Brasil

**Keywords:** Ablação por Cateter, Sistema Único de Saúde (SUS), Acesso à Saúde, Arritmias Cardíacas, Equidade em Saúde

Em um país de dimensões continentais, a facilidade de acesso ao tratamento pode ser tão determinante para o prognóstico quanto o próprio diagnóstico. Nas arritmias cardíacas, isso ganha peso especial, pois a ablação por cateter se consolidou como terapia eficaz para diversas arritmias, com resultado frequentemente superior ao tratamento farmacológico em termos de controle do ritmo, qualidade de vida, redução de hospitalizações e, em alguns casos, melhora da sobrevida. Entretanto, seu benefício populacional depende de acesso oportuno, infraestrutura adequada e financiamento coerente.^1^

A discussão sobre acesso a essa terapia no sistema público é, na prática, uma discussão sobre equidade em saúde cardiovascular. À medida que a população envelhece, a prevalência de fibrilação atrial e outras taquiarritmias aumenta, ampliando a demanda por ablação.^1^ O artigo "Ablação por radiofrequência para tratamento de arritmias cardíacas no Brasil: o cenário do Sistema Único de Saúde"^2^ aborda esse tema não de forma conceitual, como frequentemente ocorre, mas de maneira objetiva, com dados nacionais estruturados.

O trabalho oferece uma fotografia necessária e incômoda do cenário brasileiro. Com base em registros nacionais do SIH/SUS e do Cadastro Nacional de Estabelecimentos de Saúde, os autores mostram que, em 2019, foram realizados 6.471 procedimentos eletrofisiológicos no SUS. A maior parte se concentrou em intervenções de menor complexidade, como as ablações em átrio direito (53%) e as ablações simples em átrio esquerdo (22%), enquanto as ablações complexas, como as de taquicardia ventricular e de fibrilação atrial, são infrequentes. Mais do que espelhar a epidemiologia das arritmias tratadas, esse perfil expõe as limitações estruturais do sistema na oferta de procedimentos de maior complexidade.

A dimensão territorial do país aparece de forma clara nos achados de concentração regional e deslocamento. Há disparidade no número de estabelecimentos habilitados e no percentual de serviços que efetivamente realizaram procedimentos, com pior desempenho nas regiões Norte e Nordeste. Além disso, a necessidade de deslocamento intermunicipal é expressiva em diversos cenários o que acarreta atraso no tratamento, custos indiretos e risco de perda de seguimento o que impacta diretamente a efetividade terapêutica do procedimento e o impacto populacional.

Há ainda um alerta importante sobre prontidão tecnológica. Mesmo entre os serviços que realizam procedimentos, especialmente os mais complexos, nem todos dispõem de todos os equipamentos necessários, evidenciando heterogeneidade de infraestrutura. Em termos de política pública, "ter serviço" e "ter serviço pronto para o caso complexo" não são sinônimos — e essa diferença costuma se tornar mais evidente justamente nas regiões onde a oferta já é mais limitada.^3^

Se há um achado com implicações imediatas, ele está na análise econômica. Mesmo com estimativas conservadoras, o custo dos materiais excede o valor reembolsado pelo SUS em múltiplos que variam de 1,5 a 4,8 vezes, especialmente nos procedimentos de maior complexidade. Quando a conta não fecha, a consequência prática é previsível: redução de oferta, seleção de casos, necessidade de complementação local de recursos e, inevitavelmente, desigualdade de acesso. Estudos econômicos prévios já demonstraram que o custo das tecnologias utilizadas em ablação representa parcela significativa do custo total do procedimento e pode impactar diretamente a sustentabilidade da oferta no sistema público.^4^

Do ponto de vista metodológico, é importante reconhecer as limitações inerentes ao uso de bases administrativas, incluindo possíveis inconsistências de registro, ausência de dados clínicos detalhados e impossibilidade de avaliar desfechos clínicos. Ainda assim, análises populacionais têm grande valor para o planejamento em saúde, pois permitem identificar gargalos estruturais, desigualdades regionais e tendências de utilização de recursos.

O estudo também expõe uma distinção central entre eficácia e efetividade. A eficácia da ablação por cateter está solidamente demonstrada em estudos clínicos e consagrada nas diretrizes contemporâneas. O problema, no entanto, já não está na evidência. Em sistemas públicos universais, o verdadeiro desafio é fazer com que uma terapia comprovadamente eficaz deixe de ser possibilidade teórica ou privilégio localizado e se torne cuidado efetivamente disponível à população. Em outras palavras, transformar eficácia em efetividade exige que o sistema acompanhe a ciência. Isso implica rede assistencial organizada, financiamento compatível com a complexidade do procedimento, formação de profissionais especializados e manutenção de volume capaz de sustentar qualidade e segurança.

É nesse ponto que o artigo assume maior relevância. Ao invés de restringir a discussão ao mérito técnico da ablação, os autores a reposicionam onde ela de fato precisa estar, no campo do planejamento em saúde. A eletrofisiologia é uma área de alta complexidade e não se sustenta apenas com habilitação formal ou disponibilidade pontual de tecnologia. Ela depende de infraestrutura consistente, equipes experientes e escala assistencial suficiente para garantir resultados seguros e reprodutíveis. O desafio, portanto, não é apenas abrir mais centros, mas assegurar que eles tenham densidade técnica, volume, estrutura e viabilidade para funcionar de forma contínua e qualificada.

Sob essa perspectiva, o estudo aponta três frentes estratégicas incontornáveis: planejamento regional com redes de referência claramente estruturadas, sustentabilidade econômica com alinhamento mais realista entre custo e reembolso, e monitoramento por indicadores públicos capazes de expor gargalos, medir desempenho e orientar correções. Sem esses elementos, a expansão da oferta corre o risco de ser apenas nominal e em saúde pública, a tecnologia só cumpre seu papel quando deixa de ser exceção e passa a ser acesso.
