# Supplementary material for: Ablação por Cateter no SUS: Entre a Evidência Consolidada e o Acesso Limitado
Source: Arq Bras Cardiol. 2026 May 21;123(4):e20260287. [Article in Portuguese] doi: 10.36660/abc.20260287 (PMC13398883; doi:10.36660/abc.20260287)
Supplement: Translation

# Translation: Catheter Ablation in the Brazilian Unified Health System (SUS): Between Consolidated Evidence and Limited Access

**DOI:** 10.36660/abc.20260287i

**Authors:** Andre d’Avila

**Affiliations:** 1 Florianópolis SC Brazil Hospital SOS Cardio, Florianópolis, SC – Brazil

**Keywords:** Catheter Ablation, Cardiac Arrhythmias, Health Services Accessibility, Unified Health System (Brazil), Health Equity

In a country of continental dimensions, ease of access to treatment can be as crucial to prognosis as the diagnosis itself. This is especially true for cardiac arrhythmias, as catheter ablation has become an effective therapy for various arrhythmias, often yielding superior results compared to pharmacological treatment in terms of rhythm control, quality of life, reduced hospitalizations, and, in some cases, improved survival. However, its benefit to the population depends on timely access, adequate infrastructure, and coherent funding.^1^

The discussion about access to this therapy in the public system is, in practice, a discussion about equity in cardiovascular health. As the population ages, the prevalence of atrial fibrillation and other tachyarrhythmias increases, expanding the demand for ablation.^1^ The article "Radiofrequency Ablation for Treating Cardiac Arrhythmias in Brazil: Status in the Unified Health System"^2^ addresses this topic not conceptually, as often happens, but objectively, with structured national data.

This study offers a necessary and unsettling snapshot of the Brazilian healthcare landscape. Based on national records from the SIH/SUS (Hospital Information System/Unified Health System) and the National Registry of Health Establishments, the authors show that, in 2019, 6,471 electrophysiological procedures were performed within the SUS (Brazilian Unified Health System). The majority were concentrated on less complex interventions, such as right atrial ablations (53%) and simple left atrial ablations (22%), while complex ablations, such as those for ventricular tachycardia and atrial fibrillation, are infrequent. More than reflecting the epidemiology of treated arrhythmias, this profile exposes the structural limitations of the system in providing more complex procedures.

The country's territorial dimension is clearly evident in the findings regarding regional concentration and displacement. There is a disparity in the number of accredited establishments and in the percentage of services that actually performed procedures, with worse performance in the North and Northeast regions. Furthermore, the need for intermunicipal travel is significant in various scenarios, leading to delays in treatment, indirect costs, and the risk of loss of follow-up, which directly impacts the therapeutic effectiveness of the procedure and the population impact.

There is also an important warning about technological readiness. Even among services that perform procedures, especially the more complex ones, not all have all the necessary equipment, highlighting infrastructure heterogeneity. In terms of public policy, "having a service" and "having a service ready for a complex case" are not synonymous—and this difference tends to become more evident precisely in regions where the supply is already more limited.^3^

If there is one finding with immediate implications, it lies in the economic analysis. Even with conservative estimates, the cost of materials exceeds the amount reimbursed by the SUS by multiples ranging from 1.5 to 4.8 times, especially in more complex procedures. When the numbers don't add up, the practical consequence is predictable: reduced supply, case selection, the need for local resource supplementation, and, inevitably, unequal access. Previous economic studies have already demonstrated that the cost of technologies used in ablation represents a significant portion of the total cost of the procedure and can directly impact the sustainability of supply in the public system.^4^

From a methodological point of view, it is important to recognize the limitations inherent in the use of administrative databases, including possible inconsistencies in registration, lack of detailed clinical data, and the inability to assess clinical outcomes. Even so, population-based analyses are of great value for health planning, as they allow for the identification of structural bottlenecks, regional inequalities, and trends in resource utilization.

The study also highlights a key distinction between efficacy and effectiveness. The efficacy of catheter ablation is solidly demonstrated in clinical studies and enshrined in contemporary guidelines. The problem, however, is no longer the evidence. In universal public systems, the real challenge is to ensure that a demonstrably effective therapy ceases to be a theoretical possibility or a localized privilege and becomes effectively available care for the population. In other words, transforming efficacy into effectiveness requires the system to keep pace with science. This implies an organized healthcare network, funding commensurate with the complexity of the procedure, training of specialized professionals, and maintenance of a volume capable of sustaining quality and safety.

This is where the article becomes most relevant. Instead of restricting the discussion to the technical merits of ablation, the authors reposition it where it truly needs to be, in the field of healthcare planning. Electrophysiology is a highly complex area and cannot be sustained solely by formal training or the occasional availability of technology. It depends on consistent infrastructure, experienced teams, and sufficient scale of care to guarantee safe and reproducible results. The challenge, therefore, is not only to open more centers, but to ensure that they have the technical density, volume, structure, and viability to operate continuously and with high quality.

From this perspective, the study points to three unavoidable strategic fronts: regional planning with clearly structured referral networks, economic sustainability with a more realistic alignment between cost and reimbursement, and monitoring through public indicators capable of exposing bottlenecks, measuring performance, and guiding corrections. Without these elements, the expansion of supply risks being merely nominal, and in public health, technology only fulfills its role when it ceases to be the exception and becomes access.
